# Brain Activation in Response to Personalized Behavioral and Physiological Feedback From Self-Monitoring Technology: Pilot Study

**DOI:** 10.2196/jmir.8890

**Published:** 2017-11-08

**Authors:** Maxine E Whelan, Paul S Morgan, Lauren B Sherar, Andrew P Kingsnorth, Daniele Magistro, Dale W Esliger

**Affiliations:** ^1^ School of Sport, Exercise and Health Sciences Loughborough University Loughborough United Kingdom; ^2^ National Centre for Sport and Exercise Medicine Loughborough University Loughborough United Kingdom; ^3^ Medical Physics and Clinical Engineering Nottingham University Hospitals Nottingham United Kingdom; ^4^ National Institute of Health Research Leicester Biomedical Research Centre Leicester United Kingdom

**Keywords:** functional magnetic resonance imaging, neuroimaging, physical activity, sedentary behavior, interstitial glucose

## Abstract

**Background:**

The recent surge in commercially available wearable technology has allowed real-time self-monitoring of behavior (eg, physical activity) and physiology (eg, glucose levels). However, there is limited neuroimaging work (ie, functional magnetic resonance imaging [fMRI]) to identify how people’s brains respond to receiving this personalized health feedback and how this impacts subsequent behavior.

**Objective:**

Identify regions of the brain activated and examine associations between activation and behavior.

**Methods:**

This was a pilot study to assess physical activity, sedentary time, and glucose levels over 14 days in 33 adults (aged 30 to 60 years). Extracted accelerometry, inclinometry, and interstitial glucose data informed the construction of personalized feedback messages (eg, average number of steps per day). These messages were subsequently presented visually to participants during fMRI. Participant physical activity levels and sedentary time were assessed again for 8 days following exposure to this personalized feedback.

**Results:**

Independent tests identified significant activations within the prefrontal cortex in response to glucose feedback compared with behavioral feedback (*P*<.001). Reductions in mean sedentary time (589.0 vs 560.0 minutes per day, *P*=.014) were observed. Activation in the subgyral area had a moderate correlation with minutes of moderate-to-vigorous physical activity (*r*=0.392, *P*=.043).

**Conclusion:**

Presenting personalized glucose feedback resulted in significantly more brain activation when compared with behavior. Participants reduced time spent sedentary at follow-up. Research on deploying behavioral and physiological feedback warrants further investigation.

## Introduction

Physical inactivity, insufficient levels of physical activity, is attributable to 9% of premature mortality and 7% of type 2 diabetes cases [1]. In addition, sedentary behavior, defined as “any waking behavior characterized by an energy expenditure ≤1.5 metabolic equivalents of task (METs) while in a sitting or reclining posture” [2], has been strongly associated with poor cardiometabolic health [3]. With adults spending an estimated 7 hours sedentary each day [4] and the prevalence of type 2 diabetes expected to rise to 592 million by 2035 [5], it is critical to address the prevalence of physical inactivity and time spent sedentary for the amelioration of type 2 diabetes and other important chronic, noncommunicable diseases.

Over the last decade, wearable activity monitors have grown in popularity in consumer markets to help users physically track their movement behaviors (eg, active minutes, step counts, distance traveled, time spent sitting) [6,7]. Over this same time, wearable physiological sensing devices (eg, heart rate monitors, continuous glucose monitors) have been evolving and are now venturing beyond the clinical domains and into more consumer-focused markets [8]. The allure of these wearable technologies is that they provide users with real-time personalized health feedback that may act to encourage positive lifestyle behaviors (eg, moving more, sitting less, eating more healthily) [9]. However, with 32% of individuals failing to continue using these devices beyond 6 months following purchase [10], there is a need to optimize the feedback provided to the users to maintain adoption and sustain engagement with the information presented. Patel and colleagues [11] suggest that providing explanatory feedback in an understandable manner is important to encourage sustained use. Given that sustained behavior change is often poorly reported and not often achieved [12], assessing how people respond to this feedback at a cortical level (by monitoring changes in brain activation) could reveal additional insight above traditional routes such as focus groups or interviews.

Neuroimaging techniques are useful to recognize and identify the intricate relationships between cognitions, brain functions, and behavior [13]. There has been growing interest in the community toward communication neuroscience, research that provides a deep understanding of attitude and behavior change [14]. Moreover, communication neuroscience research suggests that people’s intentions and behavior are largely affected by the content and format of an advertisement [15]. One key neuroimaging tool is functional magnetic resonance imaging (fMRI), which can monitor neural responses as information is presented [16] (eg, health messages and advertisements [14,17,18]). Receiving personalized (or self-related) feedback is often associated with activation within the rostral medial prefrontal cortex (mPFC), associated with decision making and mimicry behavior [19,20], and the precuneus/posterior cingulate region, often associated with personal reflection [21-23]. In particular, self-relevant messages elucidate more activation within the mPFC than nontailored messages [24] and can predict behavior change [25]. Meta-analyses of functional neuroimaging studies also suggest that the mPFC and precuneus/posterior cingulate regions mediate self-related processing [26,27].

fMRI can improve our understanding of how cognitive processes vary between those who do change their behavior following exposure to a stimulus and those who do not subsequently change [28]. The mPFC is positioned whereby activation in this region can predict individual behavior change [14,17,29]. To date, research has largely focused on identifying neural responses to antismoking material [17,29,30] rather than diet, alcohol consumption, physical inactivity, or sedentary behavior [31]. Investigating how people respond to personalized feedback relating to these lifestyle behaviors could offer crucial insight into how best to disseminate feedback to maximize effect and thus help to design materials that optimize population health [32]. For instance, observed reductions in smoking rates have been attributed to a number of influences, in part, by the dissemination of antismoking materials (eg, cigarette packaging labels) [33]. Given that the literature to date has largely assessed how people respond to antismoking materials, fMRI may help identify how people’s brains respond to information commonly presented on the screens of wearable devices and associated smartphone apps. The authors hypothesize that the mPFC and precuneus/posterior cingulate regions will be activated given the presentation of personalized or self-relevant feedback [21-23,26,27]. The aims of this study were to identify regions of the brain activated in response to personalized behavioral and physiology feedback messages and examine behavior change and associations with levels of brain activation.

## Methods

### Participants

A total of 33 participants (57% female) were recruited from a university in the United Kingdom via advertisement posters and email. Participants were aged 30 to 60 years, had no mobility-related musculoskeletal problems, had no confirmed diagnosis of diabetes, were willing and able to comply with the study protocol, met standard fMRI safety criteria (no metal in body, not claustrophobic, not pregnant), and were right-handed. All participants completed a physical activity readiness questionnaire [34] prior to participation with positive responses assessed by a clinician.

Each participant’s consent was obtained according to the Declaration of Helsinki, and all experimental procedures were approved by the Loughborough University Ethics Advisory Committee (R15-P142).

### Procedure

**Figure 1:**
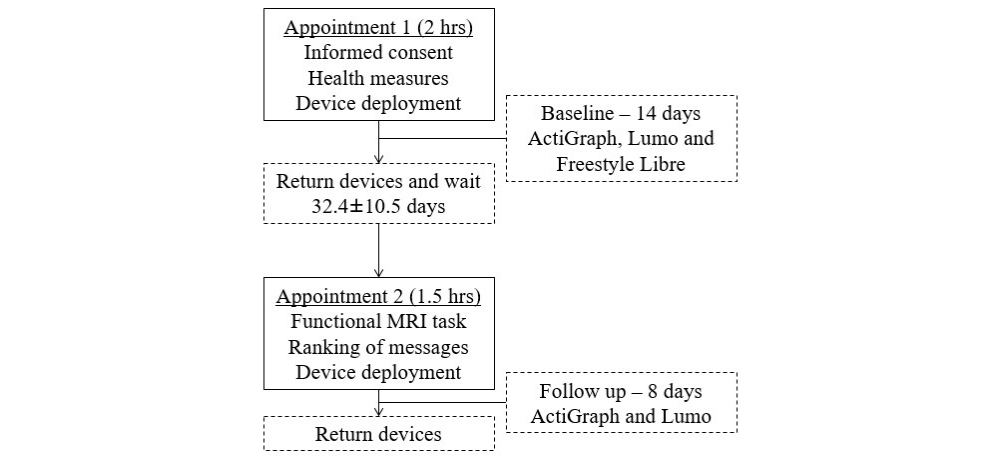
Study design.

Data were collected between June and September 2016. The study design is presented in Figure 1. During the first appointment, participants provided informed consent; answered questions relating to age, gender, ethnicity, and education; and completed a selection of health measures (body composition, blood pressure, and blood sample). Participants were fitted with 3 devices to monitor their physical activity, sedentary behavior, and glucose levels for 14 days. In addition, participants were provided an education booklet to read prior to the fMRI appointment. This booklet included background information about physical activity (eg, moderate-to-vigorous intensity [MVPA]) and also offered recommendations (eg, target range for glucose levels) to help minimize any variations in knowledge. The fMRI took place at the second appointment (on average 32.4 [SD 10.5] days following the first appointment); following this, participants continued to wear 2 devices to monitor physical activity and sedentary behavior for 8 days. At the end of the follow-up period, participants returned the devices and received a comprehensive personalized health report.

### Measures

#### Physical Health

Weight and body fat percentage were measured using the MC 780 MA scale (Tanita) following the removal of shoes and socks. Body mass index was calculated as weight (kg) divided by height (m) squared (weight/height^2^). Glucose and hemoglobin A_1c_ (HbA_1c_) were analyzed using a Cholestech LDX system and Afinion AS100 Analyzer (both Alere Inc), respectively. Participants arrived fasted for ≥8 hours prior to the collection of a capillary blood sample.

#### Accelerometry

An ActiGraph wGT3x-BT accelerometer (ActiGraph LLC) was worn on a waistband (on the right anterior axillary line) to objectively measure physical activity. Participants were asked to wear the validated device [35] during waking hours and to remove for any water-based activities (eg, showers or bathing). The accelerometry data were collected at 100 Hz resolution and integrated into 60 second epochs using ActiLife version 6.13.2 (ActiGraph LLC) and processed using Kinesoft version 3.3.80 (Kinesoft). Sedentary behavior, light activity, and MVPA were defined as ≤100 counts per min (cpm), 101 to 2019 cpm, and >2019 cpm, respectively [36]. Nonwear was identified by an interval of at least 60 consecutive minutes of zero activity intensity counts, with allowance for 1 to 2 minutes of counts between 0 and 100 [36]. Participants who had <4 valid days were excluded from analyses. A valid day was defined as having ≥10 hours of monitor wear. Accelerometers were initialized to begin monitoring at the end of appointments, which meant participants had a variable amount of possible wear on the first day. As a result, to standardize the opportunity for participants to adhere to device wear, days 2 through 8 were analyzed for both baseline and follow-up. A global wear time variable was calculated as the mean of wear time at baseline and follow-up.

#### Inclinometry

A Lumo (Lumo Bodytech Inc) posture sensor was worn on a waistband (in the lumbosacral region) in contact with the skin to measure sedentary behavior (time spent sitting, driving, lying, standing, stepping, and number of sit-to-stand transitions) during baseline and follow-up. Devices were calibrated to the wearer. Participants were asked to wear the device only during waking hours, remove it for any water-based activities (eg, showers or bathing), and place the device on charge overnight each day. The Lumo has been found to produce valid measurements of sedentary behavior compared with the ActivPAL (PAL Technologies Ltd), with a mean error of 9.5% [37]. Data from the Lumo devices were analyzed in 5-minute epochs (highest resolution) using Excel (Microsoft Corp). Nonwear was defined by 1 of 2 criteria: (1) device removal for sleep which was automatically detected if the device was placed on charge or (2) prolonged periods of the same posture deemed to be biologically implausible (ie, ≥60 minutes). Again, the Lumos were set up to begin monitoring at the end of appointments, and days 2 through 8 were analyzed for both baseline and follow-up.

#### Flash Glucose Monitoring

The Freestyle Libre flash glucose monitor (Abbott Laboratories) measures interstitial glucose levels via a minimally invasive 5 mm flexible filament inserted into the posterior upper arm. The sensor works based on the glucose-oxidase process by measuring an electrical current proportional to the concentration of glucose. Tegaderm transparent film dressing (3M Health Care) was applied on top of the sensor to maintain its position. Participants were informed not to remove the sensor and to scan at least once every 7 hours (a conservative decision as the manufacturer states 8 hours to avoid data loss). As a result, participants were able to see their real-time glucose levels during baseline wear. An indication of how many times participants viewed this information (level of exposure) was identified by the number of time they scanned. Missing data were obtained because of a fault (sensor lasted <14 days) or the participant failed to scan at least once every 8 hours. The Freestyle Libre has been previously validated against venous sampling with an overall mean absolute relative difference of 11.4% with consistent accuracy throughout the 14 days [38]. Glucose data were downloaded in 15-minute epochs (highest resolution) using Freestyle Libre version 1.0. The raw data were used to calculate the number of high glucose events (defined as ≥8.8 mmol/L) and identify valid days. Days were defined as valid if they met the prespecified threshold of ≥90% of data points (96 expected based on 4 readings each hour across each 24-hour period). All 14 days were analyzed from baseline wear. Area under the curve was calculated from the mean area of the positive peaks across the valid days using GraphPad Prism version 7.0.0 (GraphPad Software), and participants’ fasting glucose levels were used as the baseline.

### Functional Magnetic Resonance Imaging Stimuli

**Figure 2:**
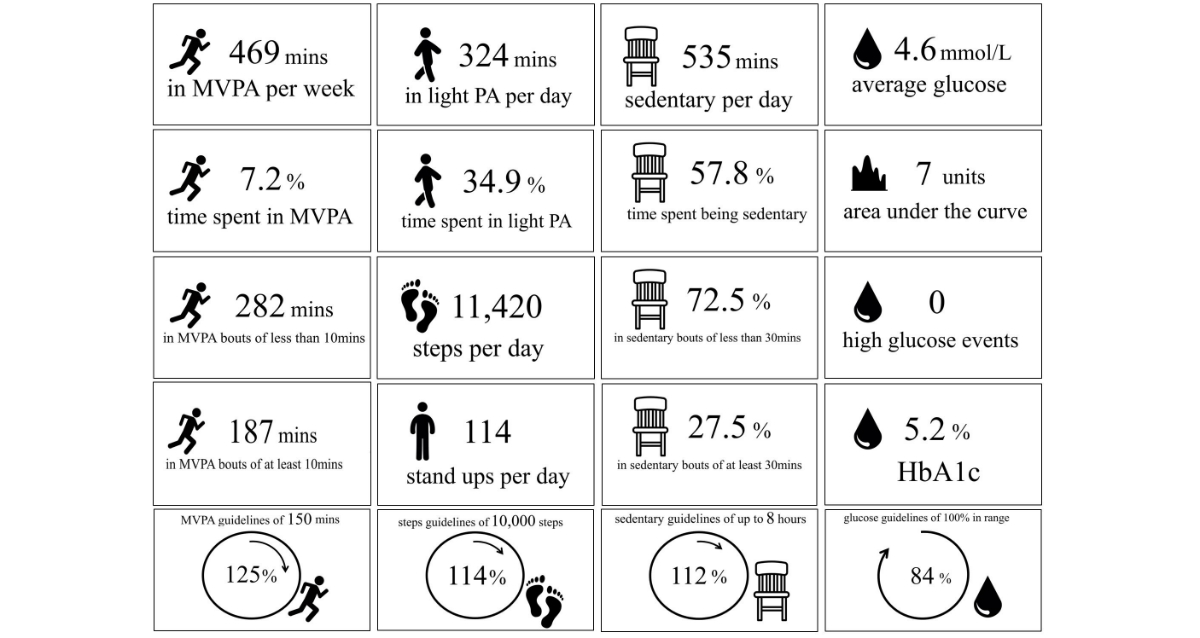
Personalized feedback stimuli.

Twenty personalized feedback messages were created for the purposes of this study and covered 4 topics: MVPA, light physical activity, sedentary behavior, and glucose levels (all presented in Figure 2 with example data). They intended to reflect feedback metrics commonly presented on wearable technologies. Data obtained via accelerometry, inclinometry, and flash glucose monitoring were analyzed and then incorporated into the personalized feedback messages. Therefore, the values presented on the messages were personalized so that the numbers varied from 1 participant to another but the image and text remained consistent. The images were matched in visual complexity, color, and text font using Axure RP Pro version 7.0.0.3190 (Axure Software Solutions Inc) to standardize the stimuli across participants. Picture icons were identified and downloaded from an icon resource website (www.flaticon.com).

**Figure 3:**
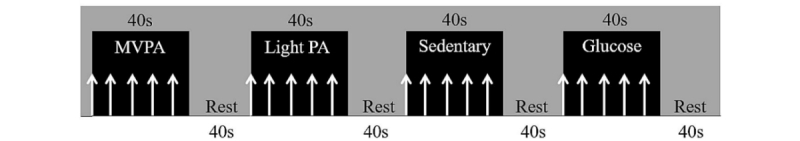
Trial setup including 8 of the 24 blocks presented.

Stimuli were presented on a monitor located 2.8 m behind the center of the scanner bore and viewed by a mirror mounted on the head coil. Adjustments to the positioning of the mirror were made for participants to ensure that the full monitor screen could be seen. We examined neural activity while participants were presented with feedback and were requested to maintain attention throughout. Prior to the start of the fMRI task, there was an initial period of 40 seconds of dummy scans which were immediately discarded. The fMRI task is outlined in Figure 3. In total, 24 blocks (12 active, 12 rest) were presented during the protocol. Each active block consisted of stimulus presentation of 5 back-to-back trials (referred to as images from this point forward) of 8 seconds each, totaling 40 seconds, followed by a rest period of 40 seconds, during which participants viewed a fixation cross and were instructed to clear their minds. The blocks and back-to-back images (within the blocks) were not presented in a counterbalanced or randomized order.

### Functional Magnetic Resonance Imaging Data Acquisition

Brain imaging data were acquired on a 3T Discovery MR750w scanner (General Electric) using a 32-channel head coil at the National Centre for Sport and Exercise Medicine, Loughborough University, United Kingdom. Structural images (T1-weighted) were acquired using a fast spoiled gradient echo (FSPGR) Bravo sequence (3D volume, FSPGR; TR=8.2 ms; TE=3.1 ms; matrix size 240×240; 160 sagittal slices; FOV=240 mm; 1 mm thick). One functional scan lasting 16 minutes (480 volumes) was acquired during the task (2D gradient echo EPI; TR=2000 ms; TE=30 ms; flip angle=75 degrees; matrix size 64×64; 35 axial slices; FOV=205 mm; 3 mm thick). Stimulus presentation and synchronization to scanner acquisition were performed using Presentation version 18.1 (Neurobehavioral Systems Inc).

### Analyses

#### Functional Magnetic Resonance Imaging Data Analysis

Functional data were preprocessed and analyzed using statistical parametric mapping (SPM12, Wellcome Department of Cognitive Neurology). All data reported are from scans that exhibited ≤3 mm in translational movement. Data were processed using a standard statistical parametric mapping approach, which consisted of scan realignment, coregistration, segmentation, normalization, and smoothing. Data were spatially aligned to the first functional image using 4th degree B-spline interpolation. Scans were then coregistered (mean functional image aligned with T1 then parameters applied to all functional images). Functional images were normalized into the Montreal Neurological Institute (MNI) standard stereotactic space with parameters applied to all functional images. A final smoothing step with a Gaussian Kernel with full width half maximum of 8 mm was applied to improve signal-to-noise ratio. The onsets and durations of each of the conditions of interest were modeled according to the block design described in the protocol. For each participant, brain activation was estimated using a general linear model (GLM) and included movement parameters (3 translations, 3 rotations) and a session constant as regressors. All regressors were convolved with SPM12’s canonical difference of the hemodynamic response function. Data were high-pass filtered with a cut off of 128 seconds to remove low-frequency noise and slow drifts in the signal. Family-wise error (FWE) correction was used to correct for multiple comparisons at  *P*_FWE_. At the first level for each participant, contrasts were computed using a series of univariate analyses of covariance (ANCOVAs), averaging activity across the topics compared with baseline: (1) MVPA>baseline, (2) light physical activity>baseline, (3) sedentary>baseline, (4) glucose>baseline, and (5) behavior>baseline. Additional contrasts were computed using a series of univariate ANCOVAs, averaging activity between the different blocks of stimuli (ie, MVPA>light physical activity and glucose>sedentary) and reverse contrasts also computed (ie, light physical activity>MVPA and sedentary>glucose).

Second level random effects models for each task were constructed that averaged across participants and were subjected to further region of interest (ROI) and between-group analysis (described below). Exploratory whole brain searches were conducted for each contrast with a threshold set at *P*<.001 and *P*<.05 for the baseline contrasts and intergroup contrasts, respectively (cluster threshold of k=0 voxels). Between-group analyses were conducted to compare gender differences and differences between those least (<150 minutes of MVPA per week) and most (≥150 minutes of MVPA per week) active. Using independent samples *t* test analysis, brain regions were labeled according to the MNI anatomic labeling tool implemented in the Wake Forest University Pickatlas (WFU Pickatlas) [39]. The average beta parameter estimates of activity during the presentation of information compared with other information blocks were extracted using MarsBaR, an ROI toolbox. All models controlled for centered demographic variables (centered age and sex). An additional centered variable (number of daily glucose scans) was included within the additional contrasts conducted.

#### Statistical Analysis

To examine demographic and self-report data, we conducted descriptive analyses using SPSS version 22.0 (IBM Corp). Two group *t* tests were conducted to produce descriptive outcomes. Repeated measures ANCOVAs were conducted to assess changes in behavior (levels of MVPA, light physical activity, and sedentary behavior) from baseline to follow-up, controlling for global wear time (average wear time). Tests of statistical significance were based on 2-sided probability (*P*<.05).

#### Correlation Analysis

Parameter estimates corresponding to each significantly activated region, identified via fMRI data analysis, were extracted for each participant. Linear regressions provided partial correlation coefficients between the parameter estimates from the significant regions of interest and subsequent behavior at follow-up (ie, time spent in MVPA, light physical activity, and sedentary), controlling for wear time. The relationships between behavior change and activity from the ROIs were examined in separate models for each ROI, and the analyses were repeated to assess behavior via both accelerometry and inclinometry data.

## Results

### Participants

**Figure 4:**
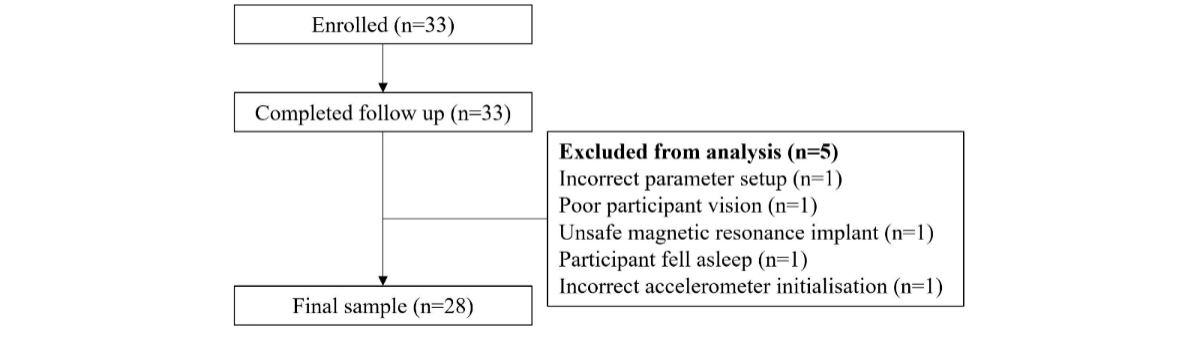
Flowchart of individuals at each stage of the study.

A flow chart of individuals through the study and the characteristics of the study sample are presented in Figure 4 and Table 1, respectively. Four participants were excluded from fMRI analyses due to incorrect scanner parameter setup, poor participant vision (without glasses), and presence of an unsafe magnetic resonance implant. One participant fell asleep, and an additional participant was excluded due to incorrect accelerometry initialization. This resulted in a final sample of 28 participants for the full study protocol.

**Table 1:** Sample characteristics.

Characteristics		Whole sample (n=28)
**Demographic**		
	Age (years), mean (SD)	44.2 (9.5)
	Male, %	42.9
**Baseline**		
	Weight (kg), mean (SD)	75.2 (15.3)
	Body mass index (kg/m^2^), mean (SD)	25.2 (4.3)
	Body fat (%), mean (SD)	26.7 (9.3)
	HbA_1c_^a^ (%), mean (SD)	5.4 (0.4)
	Glucose (mmol/L), mean (SD)	5.0 (0.6)

^a^HbA_1c_: hemoglobin A_1c_.

The 28 participants (43% male) had a mean age of 44.2 (SD 9.5) years (range 30 to 59 years). Three (11%) participants completed secondary school, 5 (18%) completed some additional training, and 20 (71%) received a bachelor’s degree or higher. Twenty-five (89%) were white, 2 (7%) were Chinese, and 1 (4%) was Asian or Asian British. Males were significantly taller (178.7 versus 167.5 cm), had a lower body fat percentage (18.8% versus 32.6%), and scanned the Freestyle Libre more frequently (9.5 versus 5.7 scans per day).

### Activated Regions of the Brain

First, we contrasted each of the 4 topics with a fixation cross. The brain regions significantly activated in response to the initial contrasts of interest are presented in Table 2. Regions include the middle and inferior occipital gyrus, middle frontal gyrus, lingual gyrus, subgyral, and thalamus (*P*<.001). No significant voxels were identified between those most and least active or between males and females.

**Figure 5:**
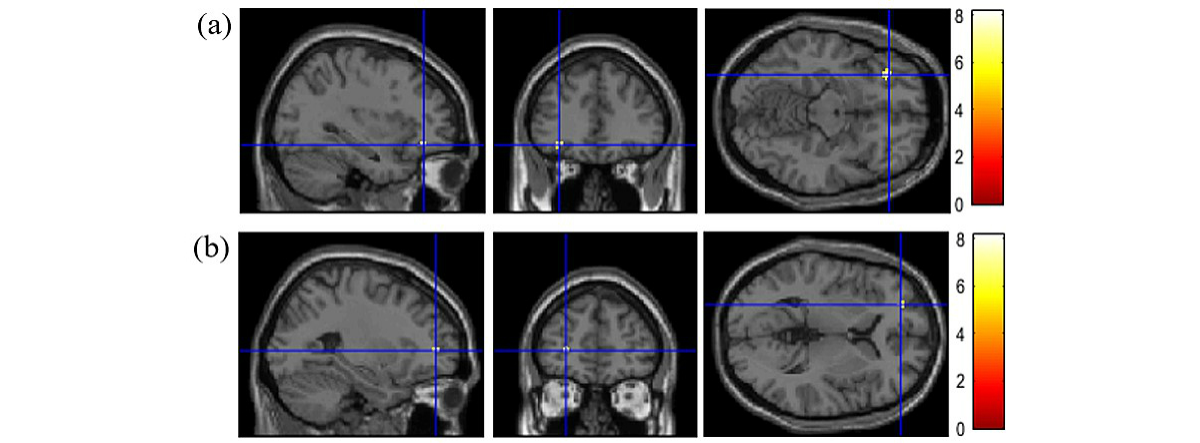
Group level significant activation pattern for the contrast glucose>behavior at the MNI coordinates (a) –32, 36, –12 and (b) –26, 48, 4.

We then proceeded to the main analysis that contrasted the topics between themselves. The brain regions identified as significantly activated are presented in Table 3. Of the additional contrasts of interest, the glucose>behavior contrast highlighted significant activation in the middle frontal gyrus (–32, 36, –12, z=5.60) and left subgyral (–26, 48, 4, z=5.33). The glucose>sedentary contrast revealed significant activation in the cuneus (–2, –80, 4, z=5.05), middle frontal gyrus (–32, 36, –12, z=4.95; –20, 34, 42, z=4.94), superior frontal gyrus (–26, 50, 4, z=4.79), and right subgyral (28, –52, 24, z=4.66) (Figure 5, Table 3).

**Table 2:** Average contrasting differences (thresholded at *P*<.001, cluster threshold of k=0 voxels).

Region		MNI^a^ coordinates														
			Hem^b^		x		y		z		Voxels		Z		t		*P*_FWE_^c^
****MVPA^d^**>baseline**																	
	Middle occipital gyrus		L		–38		–74		–14		178		6.29		9.99		<.001
	Lingual gyrus		L		–14		–94		–10		—		6.25		9.89		<.001
	Inferior occipital gyrus		L		–22		–90		–14		—		6.21		9.76		<.001
	Subgyral		R		36		–62		–16		11		6.06		9.29		<.001
	Fusiform gyrus		L		–36		–54		–16		9		5.97		9.03		<.001
	Subgyral		R		34		–84		–6		93		5.95		8.97		<.001
	Lingual gyrus		R		24		–92		–10		—		5.86		8.74		<.001
	Lingual gyrus		R		16		–90		–8		—		5.63		8.11		.001
	Inferior occipital gyrus		R		44		–76		–12		2		5.62		8.09		.001
	Middle occipital gyrus		R		30		–88		4		1		5.57		7.97		.001
**Light PA**^e^**>baseline**																	
	Cuneus		L		–16		–96		–2		101		6.47		10.61		<.001
	Middle occipital gyrus		L		–32		–84		–14		119		6.23		9.80		<.001
	Middle occipital gyrus		L		–38		–72		–14		—		6.05		9.28		<.001
	Subgyral		R		34		–84		–6		83		6.05		9.26		<.001
	Middle occipital gyrus		R		30		–84		–14		—		5.68		8.24		.001
	Middle occipital gyrus		R		46		–76		–10		23		6.01		9.14		<.001
	Subgyral		R		36		–62		–16		23		5.90		8.83		<.001
	Middle occipital gyrus		R		28		–98		6		19		5.77		8.48		<.001
	Fusiform gyrus		L		–36		–54		–16		3		5.77		8.47		<.001
	Fusiform gyrus		L		–34		–50		–18		2		5.70		8.30		<.001
	Inferior frontal gyrus		L		–54		18		20		4		5.69		8.27		<.001
	Lingual gyrus		R		16		–90		–10		10		5.65		8.18		.001
**Sedentary>baseline**																	
	Middle occipital gyrus		L		–36		–72		–16		19		5.99		9.11		<.001
	Inferior occipital gyrus		L		–38		–82		–10		46		5.95		8.98		<.001
	Subgyral		L		–20		–94		–6		36		5.87		8.77		<.001
	Middle occipital gyrus		R		36		–84		–4		4		5.78		8.50		<.001
	Inferior frontal gyrus		L		–48		14		22		3		5.65		8.16		.001
	Middle occipital gyrus		R		48		–76		–10		3		5.59		8.02		.001
	Subgyral		R		28		–88		–6		1		5.57		7.97		.001
**Glucose>baseline**																	
	Cuneus		L		–16		–96		–6		218		6.69		11.38		<.001
	Middle occipital gyrus		L		–36		–74		–16		—		6.13		9.50		<.001
	Middle occipital gyrus		L		–20		–90		–14		—		5.90		8.83		<.001
	Subgyral		R		36		–62		–16		13		5.99		9.10		<.001
	Lingual gyral		R		14		–90		–8		28		5.97		9.05		<.001
	Subgyral		R		28		–84		–6		56		5.88		8.78		<.001
	Middle frontal gyrus		L		–40		10		30		6		5.69		8.27		<.001
	Middle occipital gyrus		R		44		–76		–14		1		5.60		8.05		.001
	Middle occipital gyrus		R		30		–84		–14		2		5.58		8.00		.001
**Behavior>baseline**																	
	Middle occipital gyrus		L		–38		–72		–16		272		6.44		10.49		<.001
	Cuneus		L		–16		–96		–6		—		6.33		10.12		<.001
	Middle occipital gyrus		L		–32		–84		–14		—		6.07		9.33		<.001
	Subgyral		R		36		–62		–16		27		6.16		9.61		<.001
	Subgyral		R		34		–84		–6		135		6.14		9.53		<.001
	Lingual gyral		R		22		–92		–10		—		5.85		8.69		<.001
	Middle occipital gyrus		R		30		–84		–14		—		5.75		8.42		<.001
	Superior parietal lobule		L		–32		–62		58		5		6.06		9.28		<.001
	Middle occipital gyrus		R		46		–76		–12		24		5.96		9.00		<.001
	Middle occipital gyrus		R		48		–66		–14		—		5.88		8.79		<.001
	Fusiform gyrus		L		–36		–54		–16		8		5.95		8.98		<.001
	Middle frontal gyrus		L		–52		26		26		9		5.73		8.38		<.001
	Thalamus		R		22		–28		–2		2		5.69		8.27		.001

^a^MNI: Montreal Neurological Institute.

^b^hem: hemisphere.

^c^FWE: family-wise error.

^d^MVPA: moderate-to-vigorous physical activity.

**Table 3:** Average contrasting differences controlling for age, gender, and average daily number of glucose scans (thresholded at *P*<.05, cluster threshold of k=0 voxels).

Region		MNI^a^ coordinates							
		Hem^b^	x	y	z	Voxels	Z	t	*P*_FWE_^c^
**Glucose>behavior**									
	Middle frontalgyrus	L	–32	36	–12	25	5.60	8.17	<.001
	Subgyral	L	–26	48	4	16	5.33	7.48	<.001
**Glucose>sedentary**									
	Cuneus	L	–2	–80	4	34	5.05	6.85	<.001
	Middle frontal gyrus	L	–32	36	–12	8	4.95	6.63	<.001
	Middle frontal gyrus	L	–20	34	42	11	4.94	6.61	<.001
	Superior frontal gyrus	L	–26	50	4	3	4.79	6.29	<.001
	Subgyral	R	28	–52	24	1	4.66	6.04	<.001

^a^MNI: Montreal Neurological Institute.

^b^Hem: hemisphere.

^c^FWE: family-wise error.

**Table 4:** Behavioral characteristics derived from accelerometry and inclinometry.

	Accelerometry^a^, mean (SD)			Inclinometry^b^, mean (SD)		
	Baseline	Follow-up	*P* value	Baseline	Follow-up	*P* value
Number of valid days	7.0 (0.0)	7.0 (1.0)	—	4.2 (2.1)	5.5 (1.7)	—
Wear time	903.5 (67.7)	868.2 (70.4)	.002	924.3 (61.9)	884.0 (61.6)	.001
Step count	9065.2 (3456.2)	9634.0 (3699.3)	—	8660.9 (2995.7)	9580.3 (4326.0)	—
Counts per minute	194.0 (82.0	410.0	<.001	—	—	—
Sedentary (min)	589.0 (84.7)	560.0 (75.6)	.014	602.2 (91.1)	554.5 (89.4)	.001
Light PA^c^ (min)	265.0 (69.0)	254.2 (71.1)	—	—	—	—
Moderate (min)	45.8 (31.0)	50.7 (33.2)	—	—	—	—
Vigorous (min)	3.6 (6.6)	3.2 (6.2)	—	—	—	—
MVPA^d^ (min)	49.4 (34.2)	53.9 (35.5)	—	—	—	—
LVPA^e^ (min)	314.4 (66.4)	308.1 (72.1)	—	—	—	—
Stepping (min)	—	—	—	93.5 (26.7)	103.2 (44.1)	—
Standing (min)	—	—	—	228.5 (98.5)	226.5 (67.8)	—

^a^≥4 valid days, n=28 (100% compliance to ≥600 mins of accelerometer wear).

^b^≥1 valid day, n=23, (100% compliance to ≥600 mins of inclinometry wear).

^c^PA: physical activity.

^d^MVPA: moderate-to-vigorous physical activity.

^e^LVPA: light-to-vigorous physical activity.

### Behavior Change

The behavioral characteristics obtained via accelerometry and inclinometry are presented in Table 4. Among the 28 participants, 100% provided ≥4 days for accelerometry during baseline and follow-up. In contrast, only 15 (54%) and 20 (71%) participants provided ≥4 valid days at baseline and follow-up with the inclinometer, respectively, revealing a reduced sample (13 vs 28). As a result, the criteria for the Lumo was adjusted to ≥1 valid days. From baseline to follow-up, wear time and sedentary time reduced while minutes of MVPA and counts per minute increased. After controlling for global wear time, only time spent sedentary remained significant for both the accelerometry and inclinometry (589.0 [SD 13.9] minutes vs 560.0 [SD 11.7] minutes, *P*=.014 and 602.2 [SD 19.4] minutes vs 554.5 [SD 18.1] minutes, *P*=.001, respectively). Despite a lack of change at the whole sample level for time spent in light physical activity, MVPA, and step count, 9 (32%), 17 (61%), and 16 (57%) participants, respectively, positively increased the amount of steps, light physical activity, and MVPA at follow-up (unadjusted for global wear time). Males accumulated significantly more vigorous physical activity compared with females at baseline and follow-up (*P*=.029 and *P*=.026, respectively) and also significantly more minutes of MVPA (*P*=.033) at follow-up. No significant associations were observed between number of scans and changes in behavior via accelerometry or inclinometry (controlling for global wear time).

### Functional Magnetic Resonance Imaging Correlations

To investigate the relationship between brain activation and subsequent behavior, parameter estimates were calculated for the patterns of neural activation. Of these, only glucose feedback was positively associated with subsequent minutes of MVPA (*r*=0.392, *P*=.043). No significant associations were observed for the inclinometry data.

## Discussion

### Summary

As recent neuroimaging work has highlighted value in analyzing individual responses to feedback relating to lifestyle behaviors [14], we used fMRI to examine neural responses to personalized feedback relating to physical activity, sedentary behavior, and interstitial glucose levels. We also investigated associations between neural activity and subsequent behavior. This study lies at the intersection of 3 rapidly evolving areas of interest: wearables, lifestyle behaviors, and neuroimaging. Our study identified that presenting people with personalized feedback relating to interstitial glucose levels resulted in significantly more brain activation when compared with personalized behavioral feedback.

### Activated Regions of the Brain

Our findings identified activations within regions of the prefrontal cortex, in particular the middle frontal gyrus, subgyral, cuneus, and superior frontal gyrus upon comparison of personalized glucose feedback with behavioral feedback. Previous studies have also identified regions within the prefrontal cortex following exposure to antismoking images [29], messages encouraging sunscreen use [14], and informative nutritional labels [40]. The authors hypothesized that the mPFC and precuneus/posterior cingulate regions would be activated in our study given the presentation of personalized and self-relevant feedback [21-23,26,27]. Other fMRI studies have identified alternate activated regions including the ventromedial prefrontal cortex, inferior frontal gyrus, and amygdala when presented information about other lifestyle behaviors (eg, smoking) [30,41,42]. The findings suggest that the personalized feedback did not offer identical regions of interest when compared with the literature; however, some activation did overlap with the mPFC. Neuroimaging studies impose additional complexity because identical neural patterns can result after exposure to different stimuli [43]. However, the identified regions of brain activation may also differ because the stimuli differs between fMRI studies. Our study used a combination of text and images to inform participants about their physical activity, sedentary behavior, and interstitial glucose levels. In comparison, Falk and colleagues [41] presented images with text and numbers in a sentence (multiple lines of text) format. Overall, our findings suggest that it is possible to identify what brain regions are activated in response to personalized feedback and that glucose-related feedback evoked more brain activation. As a result, wearable technologies presenting personalized glucose feedback may be useful to employ in future interventions.

Investigating how individuals responded to personalized health-related feedback was an important component of this study as it has been well documented that receiving tailored feedback can result in greater resonance and consequently result in desirable health behaviors [44-46]. Our study demonstrated that presenting feedback pertaining to an individual’s glucose levels elicited significantly more brain activation within the middle frontal gyrus and subgyral compared with the behavioral feedback. These regions, anatomically positioned within Brodmann areas 9/10 and 47, respectively, have previously been associated with the actions of making personal moral judgments [47] and working memory [48], respectively. Previous studies have investigated messages promoting child vaccinations against measles-mumps-rubella and identified that highlighting the dangers of not vaccinating may actually be counterproductive [49]; therefore, findings are often highly dependent on the topic investigated. Future studies could investigate the role of self-affirmation, a construct suggested to increase individual sensitivity to health-risk information and incorporated in prior neuroimaging studies [29,41]. Self-affirmation essentially investigates how neural activity patterns vary to information after being exposed to personally important values (eg, friends, family, and religion). Given that the desirable outcome is for people to positively respond to health-related information, exposing a person to their personal values may provoke attention and enhance the importance of the information being given. Therefore, future investigation into whether self-affirmation could contribute to increasing the level of resonance toward personalized feedback and encourage positive behaviors may be crucial.

### Behavior Change and Associations With Brain Activation

Our study identified a significant reduction of 29 minutes (or 47 minutes using inclinometry) in time spent sedentary from baseline to follow-up. Previous findings support this finding, having observed a 39.6 minute per day reduction in time spent sedentary [50]. However, no significant differences were observed for time spent in MVPA, light physical activity, or step count. Wearable technologies research to date has offered the suggestion that people can increase their activity levels having received feedback about behavior [51,52]. However, it must be acknowledged that physical activity, for example, has been categorized as a very complex behavior and no single metric can encapsulate a person’s level of physical activity [53]. According to the literature, changes in behavior most likely occur when personalized health messages are presented in moments when action can be taken (eg, at midday to promote a walk following the consumption of lunch) [11]. Despite participants being presented personalized feedback, there are a multitude of reasons as to why they may or may not have changed their behavior during the follow-up period. Therefore, determining whether their behavior (change or no change) was because of the exposure to health-related information is truly unknown. However, emphasizing that the feedback was only briefly presented and within an unusual situation (ie, inside an MRI scanner) is warranted when comparing how people normally receive personalized feedback through wearable technologies. Further investigation could quantify or contextualize the follow-up period to try and account for extraneous variables (eg, weather, holiday, illness) or consider the inclusion of a control group to provide more definitive findings.

In regard to the relationship of activation and subsequent behavior during the follow-up period, findings identified a positive partial correlation with minutes of MVPA. Previous studies have investigated behavior change subsequent to fMRI and have demonstrated positive associations between neural response (eg, to aversive smoking-related images) and smoking cessation [17,54]. For example, Falk and colleagues [41] identified that greater reductions in sedentary behavior aligned with greater activity in the ventromedial prefrontal cortex, suggesting that if people exhibited greater levels of activation in response to the visual stimuli, those individuals were subsequently more likely to be less sedentary. On a larger scale, identifying what stimuli (ie, health messages) evoke a positive prediction of behavior (eg, being less sedentary or more active) can inform the provision of effective public health messages. It could be suggested that, despite the observed association, being presented personalized feedback about health and behavior while inside an MRI scanner is not a normal environment. Consequently, alternate neuroimaging tools could be useful for future investigation within a free-living setting. For instance, individuals could obtain personalized feedback via a wearable device or a smartphone app while their neural activity is recorded by a portable electroencephalogram system via functional near infrared spectroscopy or by eye tracking (to monitor gaze patterns and fixations). Interestingly, eye tracking has previously been conducted on various health communication materials including both cigarette advertising [55] and nutrition labels [56].

### Strengths and Limitations

Positioned at the intersection of a number of evolving interest areas, this interdisciplinary study offers a number of strengths. One strength was presenting the personalized feedback pertaining to both movement behaviors and physiology to participants. These components were objectively measured during baseline and follow-up using novel self-monitoring technologies, obtaining data to directly inform the feedback. In addition, the information that was presented in the fMRI tasks were designed based on feedback commonly presented via wearable devices or smartphone apps, reflecting what could be received in real-time in a real-world setting. Objective quantification of behavior at follow-up permitted the assessment of behavior following exposure and associations between neural activation and behavior.

Limitations of our study include the situation that participants viewed their glucose levels during baseline wear, an unavoidable situation given intentions to minimize data loss. This protocol confirms that participants had prior exposure to the glucose-related feedback subsequently presented during fMRI. However, to help try and account for this, analysis included the number of scans as a covariate because we thought the number of scans suggested the frequency with which participants viewed their glucose levels (eg, more scans equaled more exposure and so a greater awareness of their glucose levels). In addition, a lack of behavior change could be attributable to the sample that we recruited (ie, well educated and relatively healthy) and as such they could be profiled as a highly motivated audience who may not have viewed their behavior as in need of improvement. Furthermore, our unpowered sample size was another limitation, as we are unable to offer definitive interpretation of the findings. In addition, because of the number of people as active and inactive, we were unable to make any comparisons between groups of participants (eg, patterns of brain activation between those most active and least active). Finally, the pattern of neural activity observed and related psychological processes should be interpreted with caution due to the nature of reverse inference [57]. Future studies could investigate neural activity in polar groups of people classified by activity or time spent sedentary and repeat fMRI so patterns of brain activation are quantified before and after exposure to the feedback.

### Conclusion

This multidisciplinary study highlighted that fMRI can be used to assess the neural response to personalized health feedback. In particular, greater activation in the prefrontal cortex during exposure to glucose compared with behavioral feedback was observed. A reduction in time spent sedentary and a negative association between the parameter estimates and subsequent minutes of MVPA were observed. Future research deploying behavioral feedback in parallel with physiological feedback to encourage positive behavior change is warranted.

## Abbreviations

ANCOVA: analysis of covariance
cpm: counts per minute
fMRI: functional magnetic resonance imaging
FSPGR: fast spoiled gradient echo
FEW: family-wise error
GLM: general linear model
HbA1c: hemoglobin A1c
hem: hemisphere
LVPA: light-to-vigorous physical activity
MET: metabolic equivalent of task
MNI: Montreal Neurological Institute
mPFC: medial prefrontal cortex
MVPA: moderate-to-vigorous physical activity
NCSEM: National Centre for Sports and Exercise Medicine
NIHR: National Institute for Health Research
ROI: region of interest
SPM: statistical parametric mapping
WFU: Wake Forest University
