# Metadata Correction: Brain Activation in Response to Personalized Behavioral and Physiological Feedback From Self-Monitoring Technology: Pilot Study

**DOI:** 10.2196/jmir.9426

**Published:** 2017-12-21

**Authors:** Maxine E Whelan, Paul S Morgan, Lauren B Sherar, Andrew P Kingsnorth, Daniele Magistro, Dale W Esliger

**Affiliations:** ^1^ School of Sport, Exercise and Health Sciences Loughborough University Loughborough United Kingdom; ^2^ National Centre for Sport and Exercise Medicine Loughborough University Loughborough United Kingdom; ^3^ Medical Physics and Clinical Engineering Nottingham University Hospitals Nottingham United Kingdom; ^4^ National Institute of Health Research Leicester Biomedical Research Centre Leicester United Kingdom

The authors of the paper “Brain Activation in Response to Personalized Behavioral and Physiological Feedback From Self-Monitoring Technology: Pilot Study” (JMIR 2017;19(11):e384), made a mistake in the final stage of proofreading. In the affiliations list, affiliation 2 was incorrectly worded as “National Centre for Sport and Exercise Science, Loughborough University, Loughborough, United Kingdom”. Instead, this affiliation should read “National Centre for Sport and Exercise Medicine, Loughborough University, Loughborough, United Kingdom”.

The corrected article will appear in the online version of the paper on the JMIR website on December 21, 2017, together with the publication of this correction notice. Because this was made after submission to PubMed or Pubmed Central and other full-text repositories, the corrected article also has been re-submitted to those repositories.

